# Specific inhibition of FGF5-induced cell proliferation by RNA aptamers

**DOI:** 10.1038/s41598-021-82350-w

**Published:** 2021-02-03

**Authors:** Ryo Amano, Masato Namekata, Masataka Horiuchi, Minami Saso, Takuya Yanagisawa, Yoichiro Tanaka, Farhana Ishrat Ghani, Masakuni Yamamoto, Taiichi Sakamoto

**Affiliations:** 1 Department of Life Science, Faculty of Advanced Engineering, Chiba Institute of Technology, 2-17-1 Tsudanuma, Narashino-shi, Chiba, 275-0016 Japan; 2 Advangen Inc, 4-6-3 Kashiwa, Kashiwa-shi, Chiba, 277-0005 Japan; 3 Faculty of Pharmaceutical Science, Health Sciences University of Hokkaido, 1757 Kanazawa, Toubetsu, Ishikari, Hokkaido 061-0293 Japan; 4 Facility for RI Research and Education, Instrumental Analysis Center, Research Initiatives and Promotion Organization, Yokohama National University, 79-5 Tokiwadai, hodogaya-ku, Yokohama, 240-8501 Japan

**Keywords:** Biotechnology, Chemical biology

## Abstract

Fibroblast growth factor 5 (FGF5) is a crucial regulator of hair growth and an oncogenic factor in several human cancers. To generate FGF5 inhibitors, we performed Systematic Evolution of Ligands by EXponential enrichment and obtained novel RNA aptamers that have high affinity to human FGF5. These aptamers inhibited FGF5-induced cell proliferation, but did not inhibit FGF2-induced cell proliferation. Surface plasmon resonance demonstrated that one of the aptamers, F5f1, binds to FGF5 tightly (*K*_d_ = 0.7 ± 0.2 nM), but did not fully to FGF1, FGF2, FGF4, FGF6, or FGFR1. Based on sequence and secondary structure similarities of the aptamers, we generated the truncated aptamer, F5f1_56, which has higher affinity (*K*_d_ = 0.118 ± 0.003 nM) than the original F5f1. Since the aptamers have high affinity and specificity to FGF5 and inhibit FGF5-induced cell proliferation, they may be candidates for therapeutic use with FGF5-related diseases or hair disorders.

## Introduction

The fibroblast growth factor (FGF) family is involved in a wide variety of processes including cell proliferation, differentiation, and migration^1–5^. The diverse biological functions of FGFs are induced by binding to their receptors (FGFR1, FGFR2, FGFR3, and FGFR4) with the cofactor heparin sulfate^4^. The intracellular tyrosine kinase domains of the receptors are activated by FGF binding, followed the activation of various signaling pathways. FGF stimulation induces proliferation, differentiation, and migration, and FGFs are therefore involved in angiogenesis and fibrosis in several diseases and tumor growth^5^.

FGF5 is a 268-amino-acid protein (29.1 kDa) that binds with high affinity to the IIIc isoforms of FGFR1 and FGFR2, which are preferentially expressed in mesenchymal lineages^6,7^. FGF5 was first identified by the screening of transforming oncogenes^8,9^ and later characterized as a major regulator of hair growth^10–12^; FGF5 also promotes angiogenesis of human aortic endothelial cells and spermatogonial stem cell proliferation^13,14^. Therefore, development of an FGF5-specific inhibitor may be value as a hair growth enhancer and for treatment of FGF5-induced cancers; this would also contribute to understanding the mechanism of FGF5-induced signal transduction.

Aptamers are short folded nucleic acids selected from large random sequence libraries by the process known as Systematic Evolution of Ligands by EXponential enrichment (SELEX)^15,16^. Aptamers have high affinity and specificity to their various target molecules and can be used as therapeutic agents^17^. For example, an RNA aptamer specific for FGF2 exerted a strong analgesic effect in a mouse model of bone cancer pain^18^, and an anti-FGF2 aptamer also inhibited the growth of FGF2-FGFR pathway driven lung cancer cells^19^. Furthermore, the anti-FGF2 aptamer was examined for treatment of neovascular age-related macular degeneration (nAMD)^20^ and a clinical trial program has been conducted to assess the efficacy of the aptamer in patients with active nAMD.

Therefore, to develop therapeutic candidates for FGF5-related diseases or hair disorders, we performed SELEX and obtained anti-FGF5 aptamers that have high affinity and specificity to FGF5 and inhibit FGF5-induced cell proliferation. This is the first report to characterize anti-FGF5 aptamers that inhibit FGF5-iduced cell proliferation.

## Results

### Selection and identification of RNA aptamers that bind to FGF5

**Figure 1:**
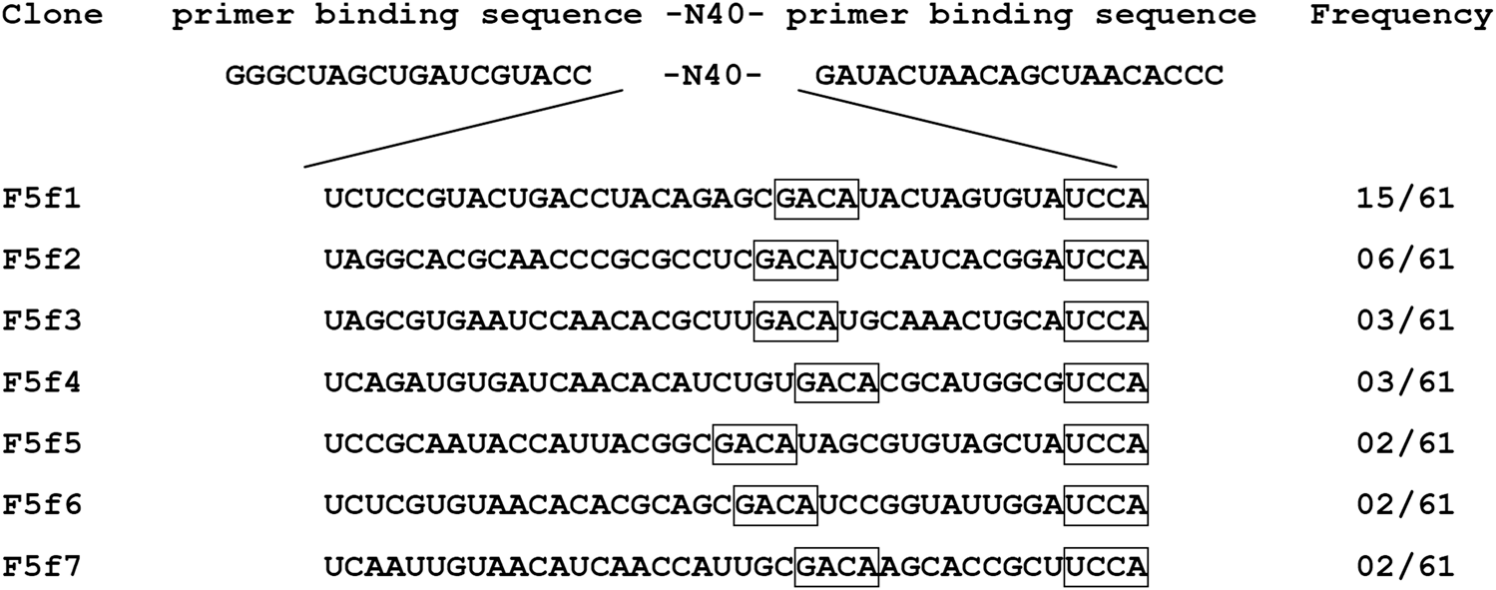
Sequence and frequency of the cloned aptamers. The selected sequences between primer binding sequences and their frequency among 61 clones are shown. The boxes indicate consensus sequences.

We first obtained RNA aptamers against human FGF5 by SELEX. An initial pool of RNAs was transcribed from a template DNA containing 40 nucleotides of random sequence. To resist ribonuclease degradation, 2′-fluoropyrimidine modifications were introduced to the RNA pool. The selection was performed against recombinant human FGF5 immobilized on nickel or cobalt charged resins. To select tightly binding aptamers, the resins were washed with buffer containing a high concentration of sodium chloride or urea with a low-molecular-weight heparin as the competitor. After seven rounds of SELEX, a total of 61 clones were randomly sequenced and seven unique sequences (F5f1–F5f7) were successfully obtained (Fig. 1). Fifteen clones contained F5f1, which was the most frequently observed sequence (15/61). Furthermore, the aptamers from F5f1 to F5f7 contained the consensus sequences 5′-GACA-3′ and 5′-UCCA-3′.

### Specific inhibition of FGF5-induced cell proliferation by aptamers

**Figure 2:**
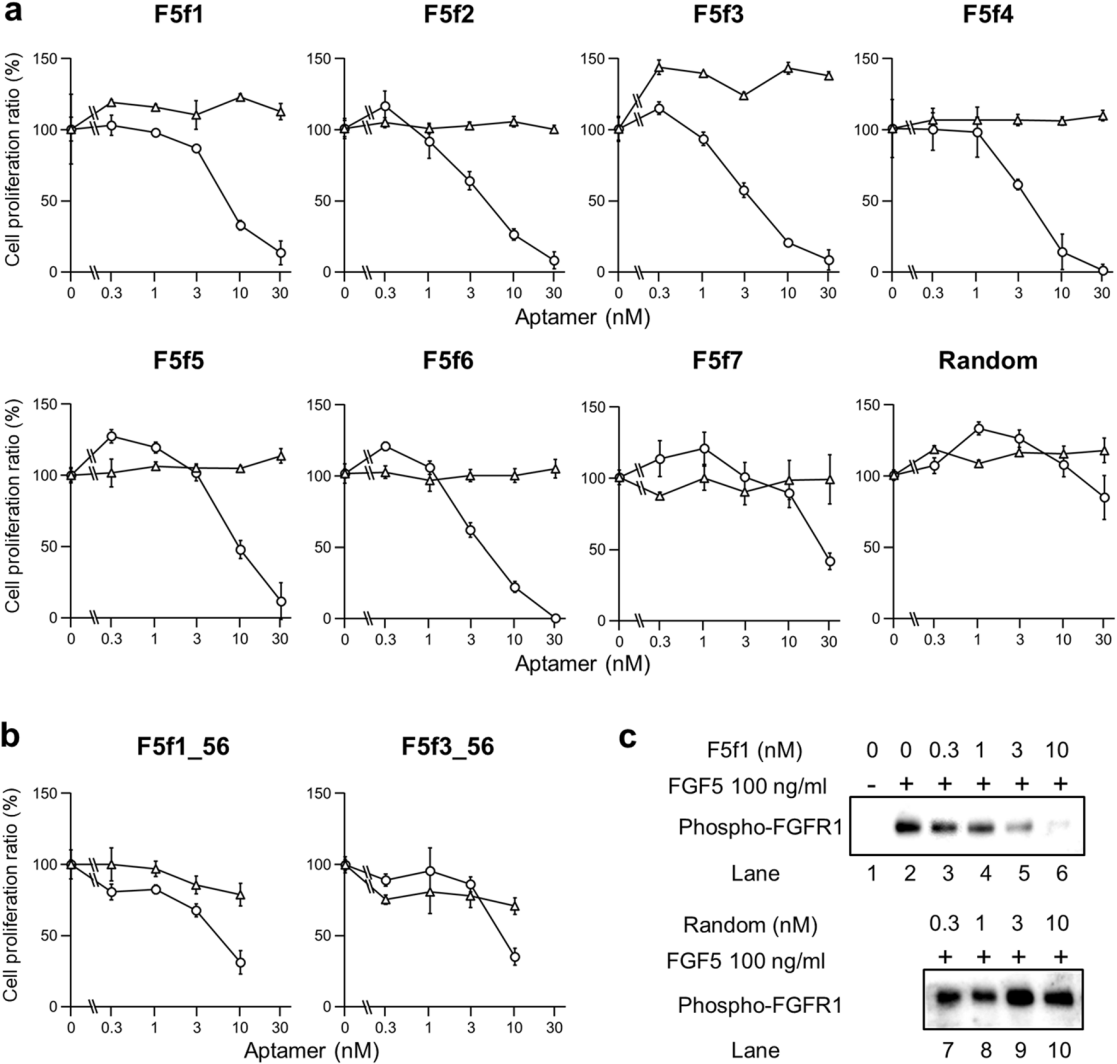
Inhibition of NIH3T3 cell proliferation by aptamers F5f1–F5f7 and random RNA (**a**) and the truncated aptamers F5f1_56 and F5f3_56 (**b**). Cells were cultured with human FGF5 (“circle”) and human FGF2 (“triangle”). The experiments were performed three times, and the mean value and errors were shown. (**c**) Immunoblotting images of phospho-FGFR1 in FGF5-stimulated NIH3T3 cells in the presence of F5f1 (lanes 3–6) or random RNA (lanes 7–10).

We assessed the inhibition of FGF5 activity as well as specificity of aptamers using a NIH3T3 cell proliferation assay. FGFR1 is expressed in NIH3T3 cells; thus, exogenous FGF5 binds to FGFR1 to induce cell proliferation^21^. Addition of aptamers with 3 nM FGF5 to NIH3T3 cells resulted in the inhibition of cell proliferation in a concentration-dependent manner (Fig. 2a), which was not observed when random RNA was used. Aptamer concentrations at which 50% cell proliferation were inhibited (IC_50_ value) were calculated (Table 1). The IC_50_ of the most frequently observed aptamer, F5f1, was 7.9 ± 0.3 nM, whereas the aptamer with the highest inhibitory activity, F5f3, was 5.2 ± 0.2 nM. On the other hand, the aptamers did not inhibit NIH3T3 cell proliferation when FGF2 was added to cells instead of FGF5. Moreover, we performed western blotting of phospho-FGFR1 and showed that the F5f1 aptamer inhibited FGFR1 phosphorylation in a dose-dependent manner, but not random RNA (Figs. 2c and S1). Therefore, the selected aptamers specifically inhibited the function of FGF5.

**Table 1:** Aptamer inhibition of NIH3T3 cell proliferation^*a*^.

Clone	IC_50_ (nM)^*b*^
F5f1	7.9 ± 0.3
F5f2	6.2 ± 1.7
F5f3	5.2 ± 0.2
F5f4	6.0 ± 2.4
F5f5	12.4 ± 0.2
F5f6	5.7 ± 0.2
F5f7	27.8 ± 0.1
F5f1_56	6.8 ± 0.8
F5f3_56	8.2 ± 1.4

^*a*^The data of the cell proliferation assay are shown in Fig. 2.

^*b*^IC_50_ is represented by the mean ± standard error from three independent measurements.

### Binding affinity and specificity of aptamers against FGF5

**Figure 3:**
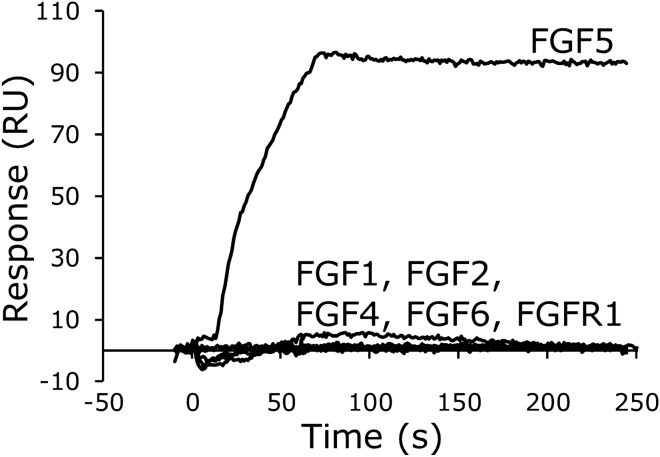
Specificity of F5f1 aptamer binding to FGF5. After immobilization of F5f1 aptamer on the sensor chip, 100 nM human FGF5, other human FGF family members, or extracellular domain of human FGFR1 was injected with four equivalents of low-molecular-weight heparin.

**Figure 4:**
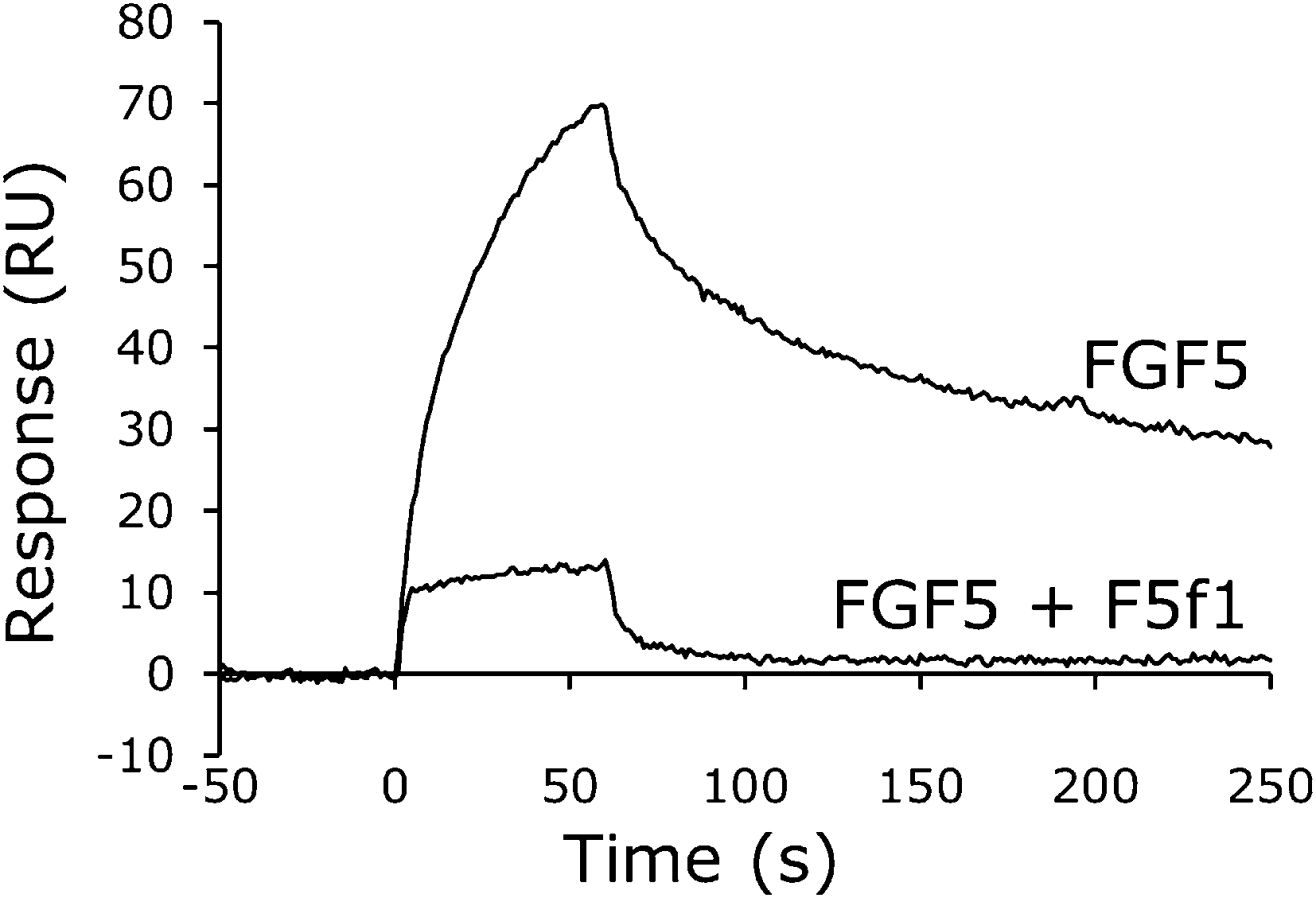
Competitive inhibition of the binding of FGF5 to FGFR1 by the F5f1 aptamer. After immobilization of the extracellular domain of human FGFR1 on the sensor chip, a mixture of 100 nM human FGF5 and 100 nM low-molecular-weight heparin was injected with or without 200 nM F5f1 aptamer.

We used surface plasmon resonance (SPR) to analyze the binding affinity of the F5f1 and F5f3 aptamers to FGF5. The dissociation constant (*K*_d_) values of F5f1 and F5f3 binding to FGF5 were 0.7 ± 0.2 nM and 0.57 ± 0.02 nM, respectively (Table 2 and Figure S2). The FGF5 specificity of F5f1 was confirmed by SPR analysis. F5f1 did not bind to FGF1, FGF2, FGF4, FGF6, or the extracellular domain of FGFR1 (Fig. 3). Furthermore, FGF5 did not bind to random RNA efficiently, although a small amount of nonspecific binding was observed (Figure S3). Similar to heparin, RNA is a highly negatively charged polymer and FGF5 has a positively charged heparin-binding site. Thus, weak binding of random RNA to FGF5 may be due to the nonspecific electrostatic interaction. Furthermore, we performed a competition assay and showed that F5f1 blocked the binding of FGF5 to FGFR1 (Fig. 4), consistent with the NIH3T3 cell proliferation assay results. Therefore, we revealed that the F5f1 aptamer specifically binds to FGF5 and competitively inhibits the binding of FGF5 to FGFR1.

**Table 2:** Kinetic parameters of aptamer binding to FGF5^*a*^.

Aptamer	*k*_on_ (× 10^5^ M^-1^ s^-1^)	*k*_off_ (× 10^–5^ s^-1^)	*K*_d_ (nM)
F5f1	3.3 ± 0.3	20 ± 6	0.7 ± 0.2
F5f3	1.7 ± 0.3	10 ± 2	0.57 ± 0.02
F5f1_56	1.8 ± 0.3	2.1 ± 0.3	0.118 ± 0.003
F5f3_56	1.36 ± 0.05	12.5 ± 0.7	0.92 ± 0.04

^*a*^The kinetic parameters are represented by the mean ± standard error from three independent measurements.

### Secondary structure prediction and truncation of F5f1 and F5f3 aptamers

**Figure 5:**
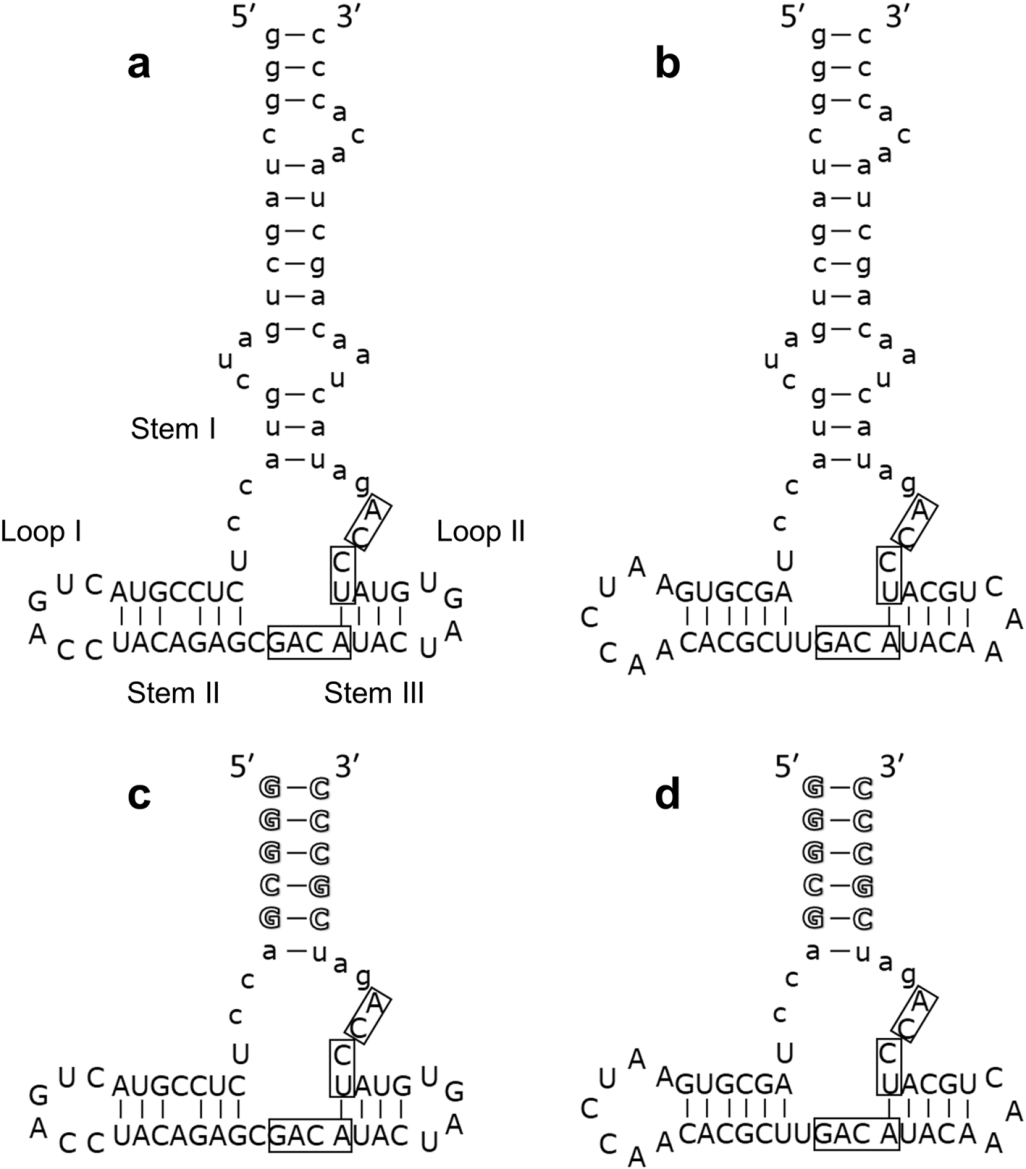
Predicted secondary structures of aptamers, F5f1 (**a**), F5f3 (**b**), F5f1_56 (**c**), and F5f3_56 (**d**). The consensus sequences are boxed. The lowercase and outline letters indicate the primer binding sequence and the added sequence, respectively.

We predicted the secondary structures of the aptamers to minimize and optimize the aptamers, using the CentroidFold program (http://rtools.cbrc.jp/centroidfold/)^22^. The aptamers have a multi-branched loop that contains the consensus sequences 5′-GACA-3′ and 5′-UCCA-3′ (Fig. 5a, b). On the basis of the predicted secondary structure, F5f1_56 and F5f3_56 (Fig. 5c, d) with 56 nucleotides (nt) were generated from F5f1 and F5f3. Five GC base pairs were added to the truncated aptamers to stabilize the stem structure. The SPR-based *K*_d_ values of FGF5 binding to F5f1_56 and F5f3_56 were 0.118 ± 0.003 nM and 0.92 ± 0.04 nM, respectively (Table 2 and Figure S2). Moreover, F5f1_56 and F5f3_56 inhibited NIH3T3 cell proliferation with IC_50_ values of 6.8 ± 0.8 nM and 8.2 ± 1.4 nM, respectively (Fig. 2b). Thus, truncated variants retained the binding activity to FGF5 and inhibitory activity of NIH3T3 cell proliferation. Clements et al. estimated that the *K*_d_ for FGF5-FGFR1 is between 0.5 and 1.5 nM according to the results of the competition assay with FGF2^23^. The *K*_d_ and IC_50_ values of the aptamers are consistent with the estimated *K*_d_ for FGF5-FGFR1 in the previous study.

## Discussion

In this work, we successfully obtained highly specific inhibitory RNA aptamers against FGF5. The aptamers from F5f1 to F5f7 had the consensus sequences 5′-GACA-3′ and 5′-UCCA-3′ (Fig. 1). Based on the predicted secondary structures of the aptamers, the consensus sequences were located in the multi-branched loop region, whereas the sequence of stem regions (Stems I and II) and apical loop regions (Loops I and II) varied (Fig. 5). This suggests that the multi-branched loop of aptamer is important for FGF5 binding. The flexible loop of aptamers is known to be important for induced fit to the surface of target proteins and binds to the proteins using weak interactions such as van der Waals contacts and hydrogen bonds^24–27^.

F5f1 aptamer bound to FGF5 with a high specificity as demonstrated by the cell proliferation assay and SPR (Figs. 2 and 3). The FGF family contains 22 identified members, which share sequence and structural similarity; therefore, it is important to ensure the specificity of the aptamer to avoid any side effects due to binding to the other FGF members. We confirmed that F5f1 did not bind to FGF4 and FGF6, which belong to the same subfamily as FGF5 in terms of sequence similarity (Figure S4). We further confirmed that the anti-FGF5 aptamers did not bind to FGF2. A clinical trial program using the anti-FGF2 aptamer has been conducted for active nAMD^19^; the sequence and secondary structure of the anti-FGF2 aptamer used in the previous study^28^ and our anti-FGF5 aptamers are different from each other (Figure S5). We also confirmed that F5f1 lacked affinity to FGFR1, which is the binding partner of FGF5. Aptamers that bind to FGFR1 or FGFR3 have been previously obtained^29–31^, and dimerized aptamers against FGFR1 or FGFR3 functioned as activators such as FGFs^30,31^. The sequences of these aptamers and our anti-FGF5 aptamers also differ from each other (Figure S5). Therefore, we expect that the anti-FGF5 aptamers will exhibit fewer side effects when used as therapeutic agents.

FGF5 was originally identified as a transforming proto-oncogene^8^. Expression of FGF5 is increased in pancreatic cancer and associated with the occurrence and metastasis of pancreatic cancer^32^. Increased FGF5 expression was also observed in cell lines from renal cell carcinoma, prostate cancer, and breast cancer^33^, and overexpression of FGF5 in melanoma cells enhanced malignancy in vitro and *in vivo*^34^. Abnormal expression of FGF5 has been observed in non-small cell lung cancer (NSCLC) tissues. NSCLC cell lines exhibited high FGF5 expression and silencing of FGF5 in NSCLC cells inhibited cell proliferation and induced cell apoptosis^35^. FGF5 is also significantly upregulated in osteosarcoma (OS) tissues and cells. Knockout of FGF5 inhibited OS cell proliferation and tumor growth in a nude mouse model, and the addition of exogenous recombinant FGF5 to OS cells promoted cell proliferation while inhibiting cell apoptosis^36^. Moreover, FGF5 was identified as a direct target of the tumor suppressive microRNA (miR) miR-188-5p in hepatocellular carcinoma^37^; miR-567 also suppressed the cell proliferation and metastasis by targeting FGF5 in OS^38^. These studies indicate that FGF5 exerts oncogenic activity in several human cancer tissues and cells, and that anti-FGF5 aptamers might inhibit these FGF5-related cancers.

FGF5 is also known as one of the crucial regulators of the hair growth cycle^10–12^. This consists of three distinct sequential phases, anagen, catagen, and telogen. Anagen is the active phase of hair growth; catagen is the regression phase where hair elongation declines; and telogen is the resting phase where elongation stops completely and eventually progresses to hair loss. FGF5 is produced in the outer root sheath of the hair follicle in the late anagen dominantly and binds to FGFR1 of the dermal papilla cells, where it induces the transition from the anagen to catagen^39,40^.

Mutations in the *FGF5* gene were identified from the angora phenotype in mice^10^ and trichomegaly in humans^11^, indicating that FGF5 terminates hair elongation consequently by switching from anagen to catagen. Therefore, inhibition of FGF5 activity by the aptamers may contribute to an extended anagen phase, resulting in promotion of hair growth and reduction of hair loss. In fact, a decapeptide showing selective inhibition of FGF5-binding with FGFR1 against FGF2-binding on NIH3T3 cells did recover coat hair growth that was suppressed by FGF5 administration *in vivo*^21^.

In this study, we obtained selected aptamers that specifically bind to FGF5 and do not bind to FGF1, FGF2, and subfamilies FGF4 and FGF6; these aptamers also inhibit FGF5-induced cell proliferation competitively. Furthermore, we succeeded in truncating the aptamer to 56 nucleotides. Therefore, these aptamers have the potential to be therapeutic agents for FGF5-associated cancers and hair loss.

## Materials and methods

### Construction of the expression plasmid for hFGF5

The DNA fragment coding residues 1–119 of *Bombyx mori* ß-1,3-glucan recognition protein (GRP) and the cleavage site of HRV 3C protease (3C) was amplified with polymerase chain reaction using KOD plus neo DNA polymerase (TOYOBO CO., LTD. Osaka, Japan) with the primers 5′-CATGCCATGGAGTACGAGGCACCACCGGC-3′ and 5′-GGAATTCCATATGCGGGCCCTGAAACAGCACTTCCAGAAATTCTACTCCTGGTGTTATTTCAGAG-3′ from pET-GRP-3C-His as a template^41^. The DNA fragment coding hFGF5 residues 21–242 [hFGF5 (21–242)] (GenBank id: NM_004464.3) was amplified with the primers 5′-CACCCATATGCACGGGGAGAAGCGTCTCG-3′ and 5′-GCCCTCGAGAGGGCTAGGTGGCTTTTTCTTTTCAG-3′ from Human Universal QUICK-Clone cDNA II (Takara Bio USA, Inc., CA, USA) as a template. DNA fragments of GRP-3C and hFGF5 (21–242) were cloned into pET-21d ( +) (Merck KGaA, Darmstadt, Germany) to give pET-GRP-3C-hFGF5 (21–242)-His.

### Construction of the expression plasmid for hFGFR1

The DNA fragment coding an artificial signal peptide Secrecon-AA^42^ was amplified using the primer 5′-GGTTTGGTGTTATCGGCGGCGGCC-3′ from the oligonucleotide 5′-ATGTGGTGGCGCCTGTGGTGGCTGCTGCTGCTGCTGCTGCTGCTGTGGCCCATGGTGTGGGCCGCCGCC-3′. The DNA fragment coding hFGFR1 residues 142–356 [hFGFR1 (142–356)] (GenBank ID NM_023110.2) was amplified using the primers 5′-GCCGCCGCCGATAACACCAAACCAAACCGTATGCC-3′ and 5′-CGCCTCCGCCCCTCTCTTCCAGGGCTTCCAG-3′ from Human Universal QUICK-Clone cDNA II (Takara Bio USA, Inc.) as a template. The DNA fragment coding Gly-Gly-Gly-Gly-Ser-His-His-His-His-His-His [G4S-His] was amplified using the primers 5′-CCTGGAAGAGAGGGGCGGAGGCGG-3′ and 5′-GATCGAACCCTTTCAATGGTGATGGTGATGG-3′ from the oligonucleotide 5′-GGCGGAGGCGGAAGCCTGGAGGTGCTGTTCCAGGGCCCCCACCATCACCATCACCATTGA-3′ that was used as a template. The DNA fragment coding pcDNA was amplified using the primers 5′-CCATCACCATTGAAAGGGTTCGATCCCTACC-3′ and 5′-CGCCACCACATGGTGGTTCGATCCTCTAGAGT-3′ from pcDNA3.4 plasmid that was used as a template (Thermo Fisher Scientific, Waltham, MA, USA). Three amplified DNA fragments of Secrecon-AA, hFGFR1 (142–356), and G4S-His were cloned into pcDNA using in vivo assembly cloning^43^. The resultant plasmid was named pcDNA-SecreconAA-hFGFR1 (142–356)-His.

### Preparation of curdlan beads

Curdlan powder (2.7 g) (FUJIFILM Wako Pure Chemical Corp., Osaka, Japan) was dissolved in 270 mL of 0.6 M NaOH and centrifuged at 4670 × *g* for 10 min at 25 °C. The soluble fraction was dispersed in 540 mL of 1-butanol at 1000 rpm with Tornado laboratory high power mixer SM-101 with a stirring blade propeller (f50 mm) (AS ONE Co., Osaka, Japan); glacial acetic acid was added until curdlan beads formed while stirring. The curdlan bead suspension was filtered with stainless steel sieves (aperture of 100 and 150 µm) and curdlan beads with a particle diameter of 100 ~ 150 µm were collected, suspended in tris-buffered saline (TBS) (10 mM Tris HCl, pH 7.5, 150 mM NaCl) as a 50% slurry, and stored at 4 °C.

### Expression and purification of hFGF5

*Escherichia coli* BL21 (DE3) pLysS competent cells were transformed with expression plasmids pET-GRP-3C-hFGF5 (21–242)-His and the transformant was inoculated directly into 2 × YT medium containing 50 µg/ml carbenicillin and 34 µg/mL chloramphenicol, and incubated at 37ºC at 120 rpm until the absorbance at 600 nm was 1.3. Expression of GRP-3C-hFGF5 (21–242)-His protein was induced by addition of isopropyl ß-D-1-thiogalactopyranoside to a final concentration of 0.1 mM. The culture was incubated at 16 °C for 24 h with gentle agitation at 90 rpm. Cells were resuspended in lysis buffer (50 mM sodium phosphate, pH 8.0, 300 mM NaCl, 10 mM imidazole, 5 mM NaN_3_) containing 1 mg/mL lysozyme (Sigma-Aldrich Co. LLC., Missouri, USA) and disrupted using sonication two times on ice for 3 min (5-s pulse, 10-s pause, 80% amplitude) using a Vibra-Cell Processor VCX-130 (Sonics & Materials, Inc., Newtown, CT, USA) equipped with 6 mm probe; subsequently, 1% of Triton X-100 was added to the cell lysate. The cell lysate was cleared by centrifugation and loaded onto a Ni–NTA superflow (QIAGEN, Hilden, Germany) affinity column (10 mm id × 100 mm). The column was washed with 20 column volume of wash buffer (50 mM sodium phosphate, pH 8.0, 300 mM NaCl, 20 mM imidazole, 5 mM NaN_3_) and GRP-3C-hFGF (21–242)-His was eluted with 5 column volume of elution buffer (50 mM sodium phosphate, pH 8.0, 300 mM NaCl, 250 mM imidazole, 5 mM NaN_3_). The eluate was mixed with the curdlan beads by a rotator for 1 h at 4 °C. Complexes of GRP-tagged protein with curdlan beads were washed with 20 column volume of TBS and were treated with GST-HRV 3C protease for 35 h at 4 °C to release the hFGF5 (21–242)-His from the curdlan beads. The curdlan bead supernatant was loaded onto TOYOPEARL AF-Heparin HC-650 M (Tosoh Corp., Tokyo, Japan) affinity column (10 mm id × 100 mm). The column was washed with 20 column volume of 20 mM HEPES–NaOH, pH 7.5, 300 mM NaCl, and then hFGF5 (21–242)-His was eluted with a 20 column volume linear gradient from 300 to 3000 mM NaCl in 20 mM HEPES–NaOH, pH 7.5. Fractions containing hFGF5 (21–242)-His protein were concentrated using ultrafiltration with the use of Amicon Ultra 4 centrifugal filtration device (10 kD molecular mass cut off) (Merck KGaA) at 4000 × *g* at 4ºC and was further purified by Superdex 200 10/200GL (Cytiva, Marlborough, MA, USA) size-exclusion chromatography column equilibrated with 20 mM HEPES–NaOH, pH 7.5, 1 M NaCl.

### Expression and purification of hFGFR1

pcDNA-SecreconAA-hFGFR1 (142–356)-His (50 µg) was transfected to 3 × 10^6^ Expi 293F cells, and hFGFR1 proteins were expressed using the Expi293 Expression System (Thermo Fisher Scientific) according to the manufacturer’s protocol. The culture medium (60 mL) that contained secreted hFGFR1 was dialyzed in 3 L of lysis buffer for 17 h at 4ºC. After dialysis, the medium was mixed with 500 µL of Ni–NTA superflow resin using a rotator for 1 h at 4ºC. The resin was transferred into the disposable column and washed with 60 mL of wash buffer. hFGFR1 proteins were eluted with 3 mL elution buffer from the Ni–NTA resin, and the eluted fraction was diluted with two volumes of TBS. Diluted hFGFR1 was concentrated using Amicon Ultra 4 centrifugal filter devices and further purified using Superdex 200 10/200GL size-exclusion chromatography column equilibrated with TBS.

### SELEX

The DNA template for the initial pool was 5′-GGGTGTTAGCTGTTAGTATC-40 N-GGTACGATCAGCTAGCCCTATAGTGAGTCGTATTA -3′ (GeneDesign, Inc., Osaka, Japan), where the T7 promoter sequence is underlined and 40 N indicates 40 nucleotides (nt) of randomized sequence. Primers 1 and 2 were 5′-TAATACGACTCACTATAGGGCTAGCTGATCGTACC-3′, 5′- GGGTGTTAGCTGTTAGTATC-3′, respectively (Hokkaido System Science Co., Ltd., Sapporo, Japan). The pool of RNAs with 2′-fluoropyrimidine modifications was created by in vitro transcription of the randomized DNA template using the Y639F mutant T7 RNA polymerase^44^ to incorporate 2′-fluoro modified pyrimidines. SELEX procedure against hFGF5 (21–242)-His was performed as previously described with minor modification^25^ Briefly, RNA was incubated with resin-immobilized hFGF5 (21–242)-His in binding buffer (20 mM Tris HCl pH 7.4, 200 mM sodium chloride, 5 mM magnesium chloride, 5 mM calcium chloride, 0.05% TWEEN20, 10% glycerol) supplemented with 10 − 100 µM low-molecular-weight heparin (approximately 5000 Da) (Dalteparin sodium; Pfizer Inc., New York, NY, USA) and 0.1 mg/mL bovine serum albumin (Sigma-Aldrich Co. LLC.). After incubation, the resin was washed five to seven times with 1 mL of washing buffer (20 mM Tris HCl pH 7.4, 500 mM sodium chloride, 5 mM magnesium chloride, 5 mM calcium chloride, 0.05% TWEEN20, 10% glycerol). Resins were finally washed three times with 1 mL of binding buffer supplemented with 3 M urea, and four times with 1 mL of washing buffer. Tightly bound RNAs were eluted from the resin via the addition of 200 µL of elution buffer (20 mM Tris HCl pH 7.4, 6 M guanidinium chloride).

### Aptamer preparation

F5f1, F5f3, F5f1_56, and F5f3_56 with 2′-fluoropyrimidine modifications were prepared as described previously^25^. Briefly, the templates of F5f1_56 and F5f3_56 were amplified from cloning vectors containing F5f1 and F5f3, respectively. All RNA samples were purified by denaturing polyacrylamide gel electrophoresis. RNA concentration was determined based on the molecular absorption coefficient at 260 nm.

### SPR experiments

SPR assays were performed as previously described using a BIAcore X instrument (Cytiva) with minor modification^25^. The 5′-biotinylated 16 mer poly dT16 oligo dissolved in SPR running buffer (20 mM Tris HCl pH 7.4, 200 mM sodium chloride, 5 mM magnesium chloride, 5 mM calcium chloride, 0.05% TWEEN20) was immobilized to both flow cell 1 and 2 on the surface of streptavidin sensor chip (Cytiva) at approximately 500 response units (RU). The aptamers containing a 16 mer poly A tail dissolved in SPR running buffer were immobilized to approximately 240 RU in flow cell 2, utilizing the hybridization of the poly A-dT. Human FGF5 (R&D Systems, Inc., Minneapolis, MN, USA) with 4 equivalents of low-molecular-weight heparin (Dalteparin sodium; Pfizer Inc.) in SPR running buffer was injected for 60 s into flow cells 1 and 2 of the sensor chip and dissociated for 180 s. The signal of flow cell 1 was subtracted from that of flow cell 2 to eliminate nonspecific interactions. Low-molecular-weight heparin was added to suppress nonspecific binding of FGF5 to the sensor chip. The sensorgrams were analyzed using BIA evaluation software (Cytiva). Dissociation constants were determined using a Langmuir (1:1) binding model. To assess the binding specificity of the aptamer, 100 nM of recombinant human FGF1, FGF2, FGF4, FGF6, or FGFR1α (IIIc)-Fc chimera (all proteins purchased from R&D Systems, Inc.) was injected with four equivalents of low-molecular-weight heparin.

For the competition assay, purified FGFR1 was dissolved in 20 mM sodium acetate (pH 6.0) and immobilized to flow cell 2 on the surface of a CM5 sensor chip (Cytiva) by the amine coupling reaction at approximately 1800 RU. Then, 100 nM human FGF5 and 100 nM low-molecular-weight heparin in SPR running buffer were injected with or without 200 nM aptamer. The signal of flow cell 1 was subtracted from that of flow cell 2 to eliminate nonspecific interactions.

### Cell culture

Murine fibroblast NIH3T3 cell (RIKEN BRC) was grown in Dulbecco’s Modified Eagle Media (DMEM) (FUJIFILM Wako Pure Chemical Corp.) supplemented with 10% fetal bovine serum (FBS, Thermo Fisher Scientific), 100 units/mL penicillin and 100 µg/mL streptomycin (FUJIFILM Wako Pure Chemical Corp.).

### Cell proliferation assay without serum

In this assay, FBS was replaced with insulin after preculture of NIH3T3 cells to prevent the enzymatic degradation of aptamers by RNases that were spontaneously contained in FBS. Briefly, NIH3T3 cells were seeded at 5,000 cells/wells in a 96-well culture plate and precultured in DMEM with 10% FBS at 37 °C in 5% CO_2_ for 24 h. Cultured cells were washed twice with phosphate-buffered saline and exchanged in DMEM supplemented with 10 ng/mL insulin, 5 μg/mL heparin (Sigma-Aldrich Co. LLC.), and 100 ng/mL human FGF5 (R&D Systems, Inc.) or 20 ng/mL human FGF2 (R&D Systems, Inc. ). Then, 0–30 nM aptamer was added to each well, and cells incubated at 37 °C in 5% CO_2_ for 48 h. Cells were counted by optical density (OD) at 450 nm using a WST-8 cell counting kit (Dojindo Molecular Technologies, Inc., Rockville, MD, USA). Inhibitory effect (IC_50_) was calculated as following formula from the obtained OD_450_ values:

$$\% \,{\text{Inhibition}} = 100 \times \left[ {{\text{OD}}_{{450}} \,{\text{in the presence of FGF5}}} \right) - \left( {{\text{OD}}_{{450}} \,{\text{in the presence of FGF5 and each aptamer}}} \right)\left] / \right[\left( {{\text{OD}}_{{450}} \,{\text{in the presence of FGF2}}} \right) - \left( {{\text{OD}}_{{450}} \,{\text{in the absence of FGF2}}} \right)]$$

The IC_50_ of each aptamer was determined by drawing an inhibition curve. The experiments were performed three times, and the mean and error were shown.

### FGFR1 phosphorylation assay

NIH3T3 cells were cultured until semi-confluent growth was observed on a six-well plate, and the medium was replaced with serum-free DMEM. After incubation for 2 h, cells were treated with 100 ng/mL FGF5 and 5 μg/mL heparin and 0 to 30 nM aptamers for 1 h at 37 °C, 5% CO_2_. Cultured cells were washed and lysed on ice for 15 min with 100 μL of RIPA buffer (Cell Signaling Technologies, Inc., Danvers, MA, USA) supplemented with protease and phosphatase inhibitor. Cell lysates were centrifuged at 21,500 × *g* for 10 min, and the supernatants were mixed with 4 × Laemmli sample buffer (Bio-Rad Laboratories, Inc., Hercules, CA, USA). Cell lysate mixtures were subjected to SDS-PAGE and western blotting using antibodies to FGFR1 or phospho-FGFR1 (Cell Signaling Technologies, Inc.). The protein concentration was determined by the BCA assay.

## Supplementary Information

Supplementary figures
