# Supplementary material for: Specific inhibition of FGF5-induced cell proliferation by RNA aptamers
Source: Sci Rep. 2021 Feb 3;11:2976. doi: 10.1038/s41598-021-82350-w (PMC7858594; doi:10.1038/s41598-021-82350-w)
Supplement: Supplementary file 1 — Supplementary figures [file 41598_2021_82350_MOESM1_ESM.pdf]

Supplementary Information

Specific Inhibition of FGF5-induced Cell Proliferation by RNA Aptamers

Ryo Amano[1], Masato Namekata[2], Masataka Horiuchi[3], Minami Saso[1], Takuya Yanagisawa[1], Yoichiro Tanaka[4], Farhana Ishrat Ghani[2], Masakuni Yamamoto[2], Taiichi Sakamoto[1*]

Corresponding Author:

* Taiichi Sakamoto[1]

Department of Life Science, Faculty of Advanced Engineering, Chiba Institute of Technology, 2-17-1 Tsudanuma, Narashino-shi, Chiba 275-0016, Japan

Telephone: +81-47-478-0317;

Fax: +81-47-478-0317;

This file includes:

Figure S1. Immunoblotting image of phospho-FGFR1 in FGF5-stimulated NIH3T3 cells in the presence of F5f1 (lanes 3–6) or random RNA (lanes 7–10).

Figure S2. SPR sensorgrams of FGF5 binding to the aptamers.

Figure S3. SPR sensorgrams of nonspecific binding of FGF5 to the random RNA.

Figure S4. Pairwise sequence alignment of FGF5 and FGF4 (a), FGF6 (b), FGF1 (c) or FGF2 (d).

Figure S5. Sequences and predicted secondary structures of aptamers against FGF2, FGFR1, and FGFR3.

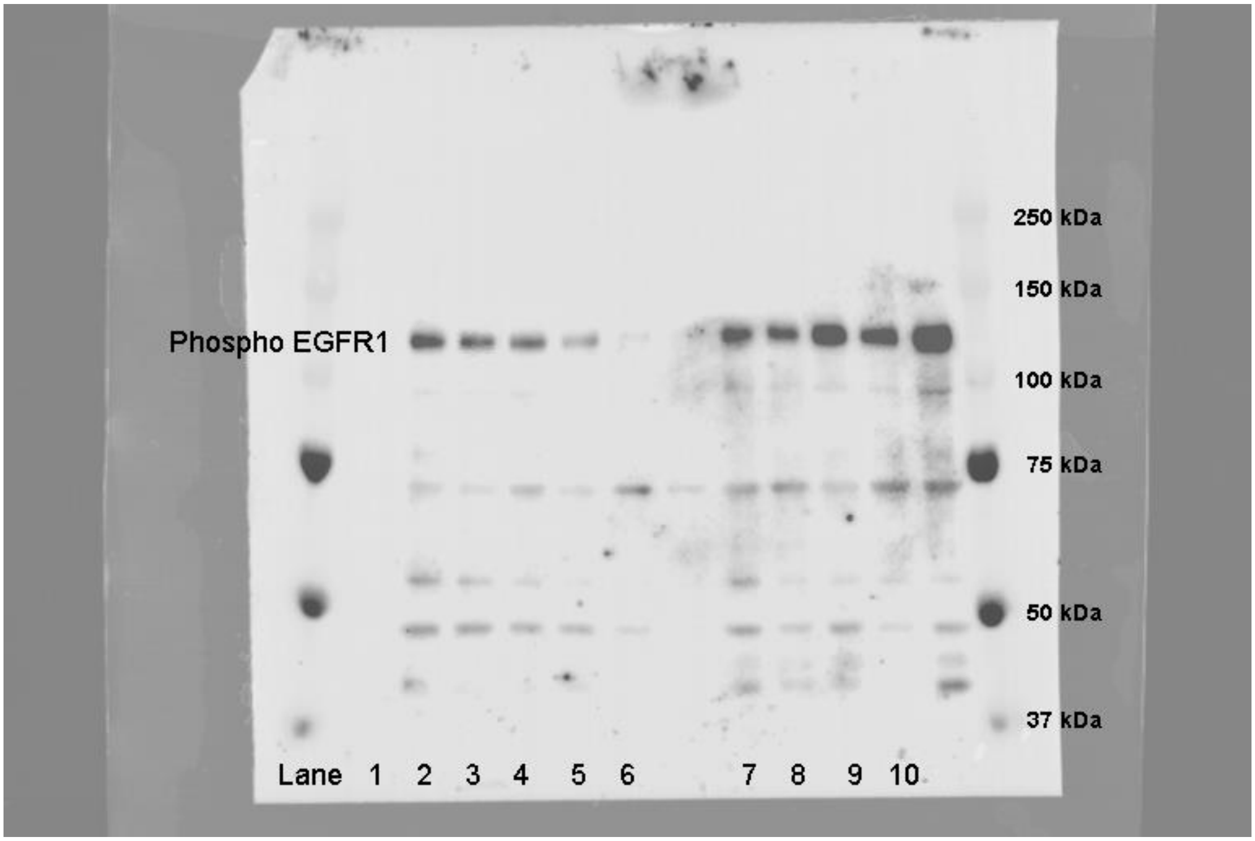

Figure S1. Immunoblotting image of phospho-FGFR1 in FGF5-stimulated NIH3T3 cells in the presence of F5f1 (lanes 3–6) or random RNA (lanes 7–10).

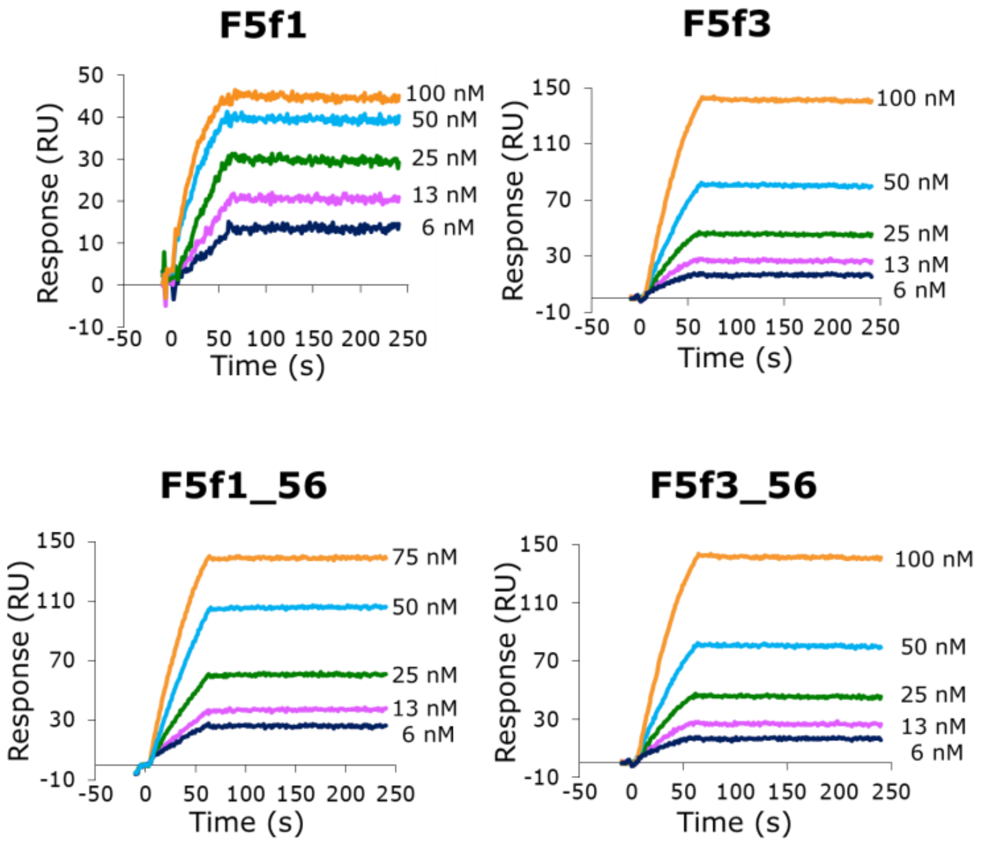

Figure S2. SPR sensorgrams of FGF5 binding to the aptamers. The aptamers F5f1, F5f3, F5f1_56, or F5f3 were immobilized on the sensor chips and various concentrations of FGF5 in SPR running buffer was injected for 60 s and dissociated for 180 s.

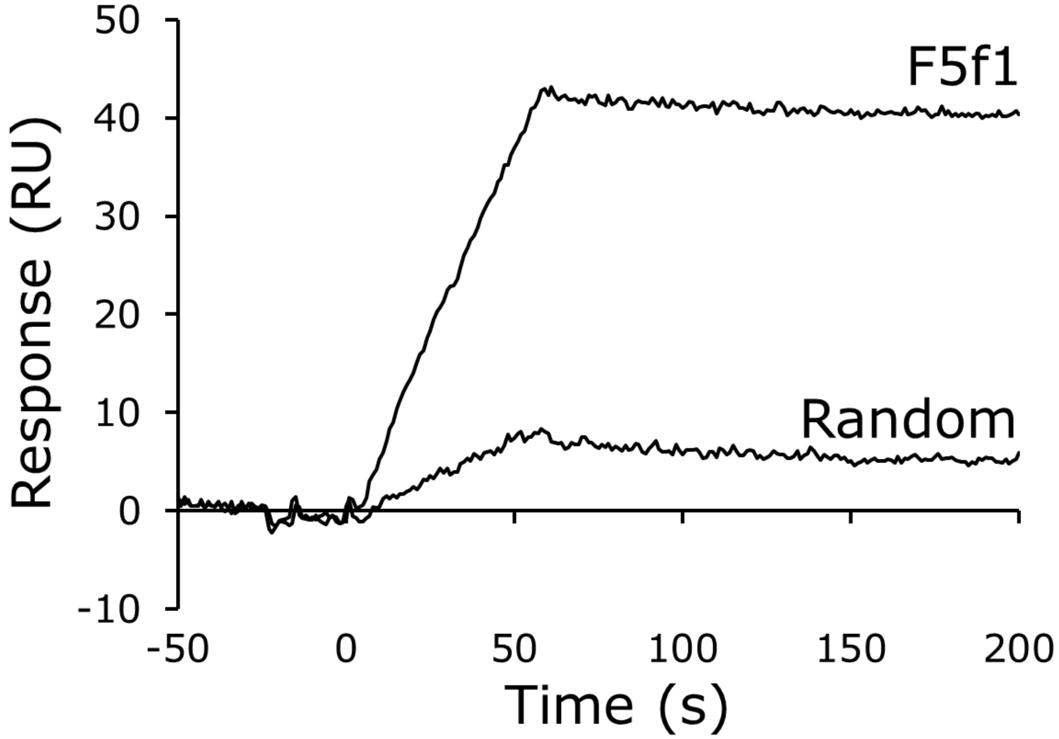

Figure S3. SPR sensorgrams of nonspecific binding of FGF5 to the random RNA. The random RNA pool and aptamers were immobilized to approximately 100 RU on the sensor chip and FGF5 in SPR running buffer was injected for 60 s and dissociated for 140 s.

a

Identity 29.0%
Similarity 40.3%

```
FGF5          1 --------MSLSFLLLLFFSHLILSAWAHGEKRLAPKGQPGPAATDRNPR     42
                        ::|...:||    .:|:.||         |: |.||....|.
FGF4          1 MSGPGTAAVALLPAVLL---ALLAPWA----------GR-GGAAAPTAPN     36

FGF5         43 GS-----SSRQSSSSAMSSS--SASSSPAASLGSQGSGLEQSSFQWSPSG     85
                |:     ..|..|..|:|.:  ..::.|..:....|:|      .:....
FGF4         37 GTLEAELERRWESLVALSLARLPVAAQPKEAAVQSGAG------DYLLGI     80

FGF5         86 RRTGSLYCRVGIGFHLQIYPDGKVNGSHEANMLSVLEIFAVSQGIVGIRG    135
                :|...|||.||||||||..|||::.|:|.....|:||:..|.:|:|.|.|
FGF4         81 KRLRRLYCNVGIGFHLQALPDGRIGGAHADTRDSLLELSPVERGVVSIFG    130

FGF5        136 VFSNKFLAMSKKGKLHASAKFTDDCKFRERFQENSYNTYASAIHRTEKTG    185
                |.|..|:|||.||||:.|..|||:|.|:|....|:||.|.|..:
FGF4        131 VASRFFVAMSSKGKLYGSPFFTDECTFKEILLPNNYNAYESYKY------    174

FGF5        186 REWYVALNKRGKAKRGCSPRVKPQHISTHFLPRFKQSEQPELSFTVTVPE    235
                ...::||:|.||.|:|  .||.|....||||||.
FGF4        175 PGMFIALSKNGKTKKG--NRVSPTMKVTHFLPRL----------------    206

FGF5        236 KKKPPSPIKPKIPLSAPRKNTNSVKYRLKFRFG    268

FGF4        207 ---------------------------------    206
```

b

Identity 30.2%
Similarity 40.6%

```
FGF5          1 MSLSFLLLLFFS----HLILSAWAHGEKRLAPKG------QPGPAATDRN     40
                |:|...|.:..|    .|..:.||     |...|      .|.||.|..|
FGF6          1 MALGQKLFITMSRGAGRLQGTLWA-----LVFLGILVGMVVPSPAGTRAN     45

FGF5         41 -----PRGSSSRQSSSSAMSSSSASSSPAASLGSQGSGLEQSSFQWSPSG     85
                     .||..:..|.|.|                 |...|.:...|. ||
FGF6         46 NTLLDSRGWGTLLSRSRA-----------------GLAGEIAGVNWE-SG     77

FGF5         86 -----RRTGSLYCRVGIGFHLQIYPDGKVNGSHEANMLSVLEIFAVSQGI    130
                     :|...|||.||||||||:.|||:::|:||.|..|:|||..|.:|:
FGF6         78 YLVGIKRQRRLYCNVGIGFHLQVLPDGRISGTHEENPYSLLEISTVERGV    127

FGF5        131 VGIRGVFSNKFLAMSKKGKLHASAKFTDDCKFRERFQENSYNTYASAIHR    180
                |.:.||.|..|:||:.||:|:|:..|.::|||||....|:||.|.|.:::
FGF6        128 VSLFGVRSALFVAMNSKGRLYATPSFQEECKFRETLLPNNYNAYESDLYQ    177

FGF5        181 TEKTGREWYVALNKRGKAKRGCSPRVKPQHISTHFLPRFKQSEQPELSFT    230
                ..      |:||:|.|:.|||  .:|.|....||||||.
FGF6        178 GT------YIALSKYGRVKRG--SKVSPIMTVTHFLPRI-----------    208

FGF5        231 VTVPEKKKPPSPIKPKIPLSAPRKNTNSVKYRLKFRFG    268

FGF6        209 --------------------------------------    208
```

Figure S4. Pairwise sequence alignment of FGF5 and FGF4 (a), FGF6 (b), FGF1 (c) or FGF2 (d). Sequence alignments were performed using EMBL-EBI search and sequence analysis tool (https://www.ebi.ac.uk/Tools/psa/)[1].

c

Identity 20.7%
Similarity 30.0%

```
FGF5   1 MSLSFLLLLFFSHLILSAWAHGEKRLAPKGQPGPAATDRNPRGSSSRQSS  50

FGF1   1 --------------------------------------------------   0

FGF5  51 SSAMSSSSASSSPAASLGSQGSGLEQSSFQWSPSG-RRTGSLYCRVGIGF  99
            |:....::..|.:          ..|...|.. ::...|||..| |.
FGF1   1 ---MAEGEITTFTALT----------EKFNLPPGNYKKPKLLYCSNG-GH  36

FGF5 100 HLQIYPDGKVNGSHEANMLSV-LEIFAVSQGIVGIRGVFSNKFLAMSKKG 148
         .|:|.|||.|:|:.:.:...: |::.|.|.|.|.|:...:.::|||...|
FGF1  37 FLRILPDGTVDGTRDRSDQHIQLQLSAESVGEVYIKSTETGQYLAMDTDG  86

FGF5 149 KLHASAKFTDDCKFRERFQENSYNTYASAIHRTEKTGREWYVALNKRGKA 198
         .|:.|....::|.|.||.:||.||||.|..| .||   .|:|.|.|.|..
FGF1  87 LLYGSQTPNEECLFLERLEENHYNTYISKKH-AEK---NWFVGLKKNGSC 132

FGF5 199 KRGCSPRVKPQHISTHFLPRFKQSEQPELSFTVTVPEKKKPPSPIKPKIP 248
         |||  ||......:..|||....|:
FGF1 133 KRG--PRTHYGQKAILFLPLPVSSD------------------------- 155

FGF5 249 LSAPRKNTNSVKYRLKFRFG 268

FGF1 156 -------------------- 155
```

d

Identity 21.5%
Similarity 31.7%

```
FGF5   1 --------------------------------------------------   0

FGF2   1 MVGVGGGDVEDVTPRPGGCQISGRGARGCNGIPGAAAWEAALPRRRPRRH  50

FGF5   1 -----------------------MSLSFLLLLFFSHLILSAWAHGEKRLA  27
                                .|.|.|            ...|..|..
FGF2  51 PSVNPRSRAAGSPRTRGRRTEERPSGSRL------------GDRGRGRAL  88

FGF5  28 PKGQPGPAATDRNP----------RGSS---------SRQSSSSAMSSSS  58
         |.|:.|.....|.|          ||::         ||...:..|::.|
FGF2  89 PGGRLGGRGRGRAPERVGGRGRGRGTAAPRAAPAARGSRPGPAGTMAAGS 138

FGF5  59 ASSSPAASLGSQGSGLEQSSFQWSPSG--RRTGSLYCRVGIGFHLQIYPD 106
         .::.||.. ...|||    :|   |.|  :....|||:.| ||.|:|:||
FGF2 139 ITTLPALP-EDGGSG----AF---PPGHFKDPKRLYCKNG-GFFLRIHPD 179

FGF5 107 GKVNGSHEANMLSV-LEIFAVSQGIVGIRGVFSNKFLAMSKKGKLHASAK 155
         |:|:|..|.:...: |::.|..:|:|.|:||.:|::|||.:.|:|.||..
FGF2 180 GRVDGVREKSDPHIKLQLQAEERGVVSIKGVCANRYLAMKEDGRLLASKC 229

FGF5 156 FTDDCKFRERFQENSYNTYASAIHRTEKTGREWYVALNKRGKAKRGCSPR 205
         .||:|.|.||.:.|:||||.|..:.:      |||||.:.|:.|.|  .:
FGF2 230 VTDECFFFERLESNNYNTYRSRKYTS------WYVALKRTGQYKLG--SK 271

FGF5 206 VKPQHISTHFLPRFKQSEQPELSFTVTVPEKKKPPSPIKPKIPLSAPRKN 255
         ..|...:..|||...:|
FGF2 272 TGPGQKAILFLPMSAKS--------------------------------- 288

FGF5 256 TNSVKYRLKFRFG 268

FGF2 289 ------------- 288
```

Figure S4 (continued). Pairwise sequence alignment of FGF5 and FGF4 (a), FGF6 (b), FGF1 (c) or FGF2 (d).

a

Anti-FGF2 aptamer

5′-GGGAUACUAGGGCAUUAAUGUUACCAGUGUAGUCCC-3′

Anti-FGFR1 aptamer

5′- GGAGGGAAAAGTTATCAGGTCAAAACGTCTTTATGGCTGGGGATGGTGTG GGTTGTTTTGATTAGTTTTGGAGTACTCGCTCC-3′

Anti-FGFR3 aptamer

5′-AGCUCCAGAAGAUAAAUUACAGGCAGAGGCUGACGUAAACAGACAUU GAUGGGACCCACCCUUCCGCUGGCAACAACUAGGAUACUAUGACCCC-3′

b

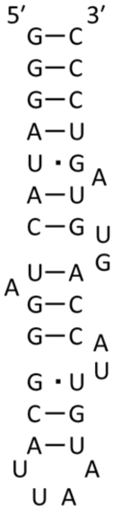

Figure S5. Sequences and predicted secondary structure of aptamers against FGF2, FGFR1, and FGFR3. (a) The sequences of the aptamers. All C and U of the anti-FGF2 aptamer and the anti-FGFR3 aptamer are 2′-fuluoro modified. The anti-FGFR1 aptamer is DNA aptamer. (b) The secondary structure of the anti-FGF2 aptamer was predicted using the CentroidFold program (http://rtools.cbrc.jp/centroidfold/)[2].

References

1. Madeira, F. *et al.* The EMBL-EBI search and sequence analysis tools APIs in 2019. *Nucleic Acids Res.* **47**, W636-W641, doi: 10.1093/nar/gkz268 (2019).
2. Sato, K., Hamada, M., Asai, K. & Mituyama, T. CENTROIDFOLD: a web server for RNA secondary structure prediction. *Nucleic Acids Res.* **37**, W277-280, doi:10.1093/nar/gkp367 (2009).
